# Landscape of immune cell gene expression is unique in predominantly WHO grade 1 skull base meningiomas when compared to convexity

**DOI:** 10.1038/s41598-020-65365-7

**Published:** 2020-06-03

**Authors:** Zsolt Zador, Alexander P. Landry, Michael Balas, Michael D. Cusimano

**Affiliations:** Division of Neurosurgery, Department of Surgery, St. Michael’s Hospital, Toronto, ON Canada

**Keywords:** Gene ontology, Gene regulatory networks, Target identification, Gene expression, Molecular medicine

## Abstract

Modulation of tumor microenvironment is an emerging frontier for new therapeutics. However in meningiomas, the most frequent adult brain tumor, the correlation of microenvironment with tumor phenotype is scarcely studied. We applied a variety of systems biology approaches to bulk tumor transcriptomics to explore the immune environments of both skull base and convexity (hemispheric) meningiomas. We hypothesized that the more benign biology of skull base meningiomas parallels the relative composition and activity of immune cells that oppose tumor growth and/or survival. We firstly applied gene co-expression networks to tumor bulk transcriptomics from 107 meningiomas (derived from 3 independent studies) and found immune processes to be the sole biological mechanism correlated with anatomical location while correcting for tumour grade. We then derived tumor immune cell fractions from bulk transcriptomics data and examined the immune cell-cytokine interactions using a network-based approach. We demonstrate that oncolytic Gamma-Delta T cells dominate skull base meningiomas while mast cells and neutrophils, known to play a role in oncogenesis, show greater activity in convexity tumors. Our results are the first to suggest the importance of tumor microenvironment in meningioma biology in the context of anatomic location and immune landscape. These findings may help better inform surgical decision making and yield location-specific therapies through modulation of immune microenvironment.

## Introduction

Meningiomas are amongst the most common adult brain tumors and constitute approximately 30% of all intracranial neoplasms^1^. Surgery remains a key part of treatment for symptomatic or growing meningiomas and outcomes are largely determined by tumor biology^2,3^ and extent of resection^4^. Approximately 70% of meningiomas have benign characteristics and the average disease-free survival is 90% over 10 years^2^, but lifetime recurrence can be as high as 50%^5^. The extent of surgical excision is largely determined by technical feasibility which is a function of anatomical location of the tumor, adherence to adjacent tissue and the eloquent structures limiting the extent of removal^6^. Achieving complete excision of meningiomas that grow around structures at the base of the skull (so-called “skull base meningiomas”) poses a particular challenge due to the proximity of neurovascular structures as well as the often narrow surgical corridors (these are contrasted from “convexity meningiomas” which grow elsewhere in the supratentorial space). Consequently, they may require longer operative times and can have lower rates of complete excision. Given the increased likelihood of being left with post-operative residual tumour, understanding the biology skull base meningiomas is of particular interest when it comes to developing new medical treatments to prevent further growth or recurrence.

Multiple studies show that skull base meningiomas are more likely to have benign biology whereas tumors with more aggressive behavior (atypical or malignant meningiomas) can constitute close to 30% of convexity/parafalcine tumors^7–9^. Recent studies have analyzed the genetic makeup of meningiomas and found recurrent mutations in the neurofibromatosis type 2 (NF2) gene and/or loss of chromosome 22 (NF2/chr22loss) to be more prevalent in the cerebral and cerebellar hemisphere^10^. The vast majority of non-NF2/chr22loss meningiomas are typically benign and tend to be located medially on the skull base. Smoothened, frizzled family receptor (SMO) mutations are also implicated in non-NF2/chr22loss and medial meningiomas through increased activation of the Hedgehog pathway^10^. Recurrent polymerase (RNA) II (DNA directed) polypeptide A (POLR2A) mutations have recently been found to classify a distinct subset of benign meningiomas with meningothelial histology and a preponderance to localize in the tuberculum sellae^11^. Analysis of methylation subtypes of meningiomas showed a relatively greater proportion of skull base lesions among the more benign subgroups with longer disease-free survival, when compared to the groups with less favourable outcomes. On the other hand, all tumors in the malignant methylation subtype were located exclusively on the convexity^8^.

Systems level analysis of gene expression data, which considers genes as units of a system rather than isolated entities, can identify key molecular processes which map to biological/clinical phenotypes^12,13^. This approach carries the distinct advantage of being unbiased in deriving meaning from high dimensional, biologically-modelled data. Gene co-expression networks are one example of such system level analysis, wherein similar genes are grouped into “modules” of similar function^14^. This technique has been successfully applied to explore complex phenotypes in Huntington’s disease^15^, peripheral nerve regeneration^16^ and weight gain^17^. It has also been used to identify relevant subgroups of meningioma^18^. In the current study we apply this technique along with another network-based approach to model the immune landscape of skull base and convexity meningiomas.

We hypothesize that different meningioma locations are associated with distinct biological mechanisms, likely related to immune cell composition and/or activity. To capture these geographical patterns in meningioma biology we analyzed the transcriptomics profiles of 107 meningiomas originating from either the skull base or convexity.

## Results

### Co-expression network and module-phenotype correlation

**Figure 1:**
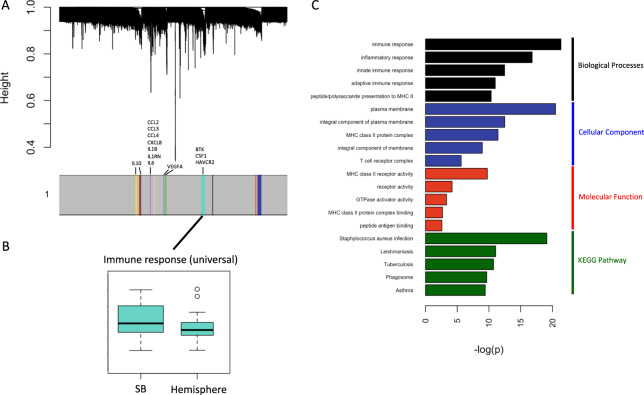
Gene co-expression network reveals immune function to correlate strongly with meningioma location. (**A**) Gene dendrogram illustrating modules. The grey denotes genes which are not implicated with any modules. Labels indicate cytokines which are positively correlated with the module meta-gene expression (Pearson correlation>0.6, p < 0.05). (**B**) Boxplot depicting the correlation between location and meta-gene expression level of the only significant module with DAVID annotations (Mann Whitney p = 0.005). As indicated in (**C**), this module maps to diverse immune processes, and is therefore labeled as “immune response (universal)”. C: Gene ontology terms ranked by Bonferroni p-value.

A gene co-expression network was constructed from 19,011 gene transcripts. Using an adaptive hierarchical clustering model^14^, we discovered fourteen gene modules (Fig. 1A). We next compared the biology of both tumor locations by correlating phenotype with meta-gene expression levels. Three modules were found to be significantly different between hemispheric and skull base meningiomas (Mann Whitney p < 0.05), only one of which annotated significantly (Bonferroni p < 0.05) to DAVID gene ontology/pathways (Fig. 1B,C). We do note that one of these modules mapped significantly to the “extracellular exosome” cellular compartment, but did not annotate to anything else in DAVID and therefore was not considered in further analysis. We also confirmed module significance by correcting the model for WHO grade, age, and sex and note that significance is preserved for all 3 modules. Finally, we selected the 10 most correlated genes to the module eigengene from the 3 modules which are significantly associated with tumour location and used these 30 genes in a logistic regression model to predict tumour location. The area under the resultant receiver-operating characteristic (ROC) curve was 0.849 (Supplemental Fig. 2).

### Immune composition of meningiomas by location

**Figure 2:**
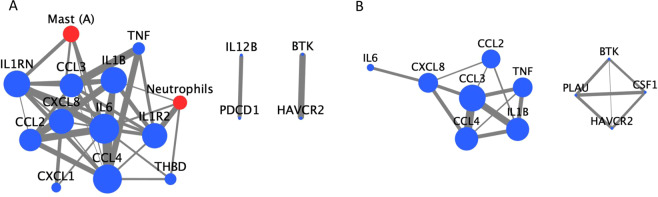
Network demonstrating the connectivity of immune cell fractions and cytokine transcriptomics in convexity (**A**) and skull base (**B**) meningiomas. Cytokines are represented in blue whereas immune cells are represented in red. Edge thickness is proportional to Pearson correlation and node size is proportional to eigenvector centrality, a measure of the influence of a particular node in the network. Notably, the eigenvector centrality ranges from 0 to 0.39 (*IL-6*) in the convexity model and from 0 to 0.50 (*CCL3*) in the skull base model. Neutrophils and activated mast cells are significantly correlated with cytokines in the convexity model using our thresholding criteria, while the skull base model contains only cytokines.

Using network analysis, we examined the biological activity of each immune cell type by correlating them with cytokine expression. On inspection of cytokine-cell networks there was a clear difference in the network configurations of convexity and skull base meningiomas (Fig. 2). Notably, activated mast cells and neutrophils are present in the convexity network with our cutoff of Pearson ρ>0.6, p < 0.05. In this network, mast cells are most correlated with *IL-6* (ρ = 0.69, p = 3.0 ×10^−6^) and neutrophils with *IL1R2* (ρ = 0.74, p = 2.4 ×10^−7^). In the skull base network, there are no cell-ligand interactions that meet our threshold, though we note that the strongest associations are between *BTK* and both plasma cells (ρ = 0.57, p = 2.4 ×10^−7^) and monocytes (ρ = 0.54, p = 1.3 ×10^−6^).

**Figure 3:**
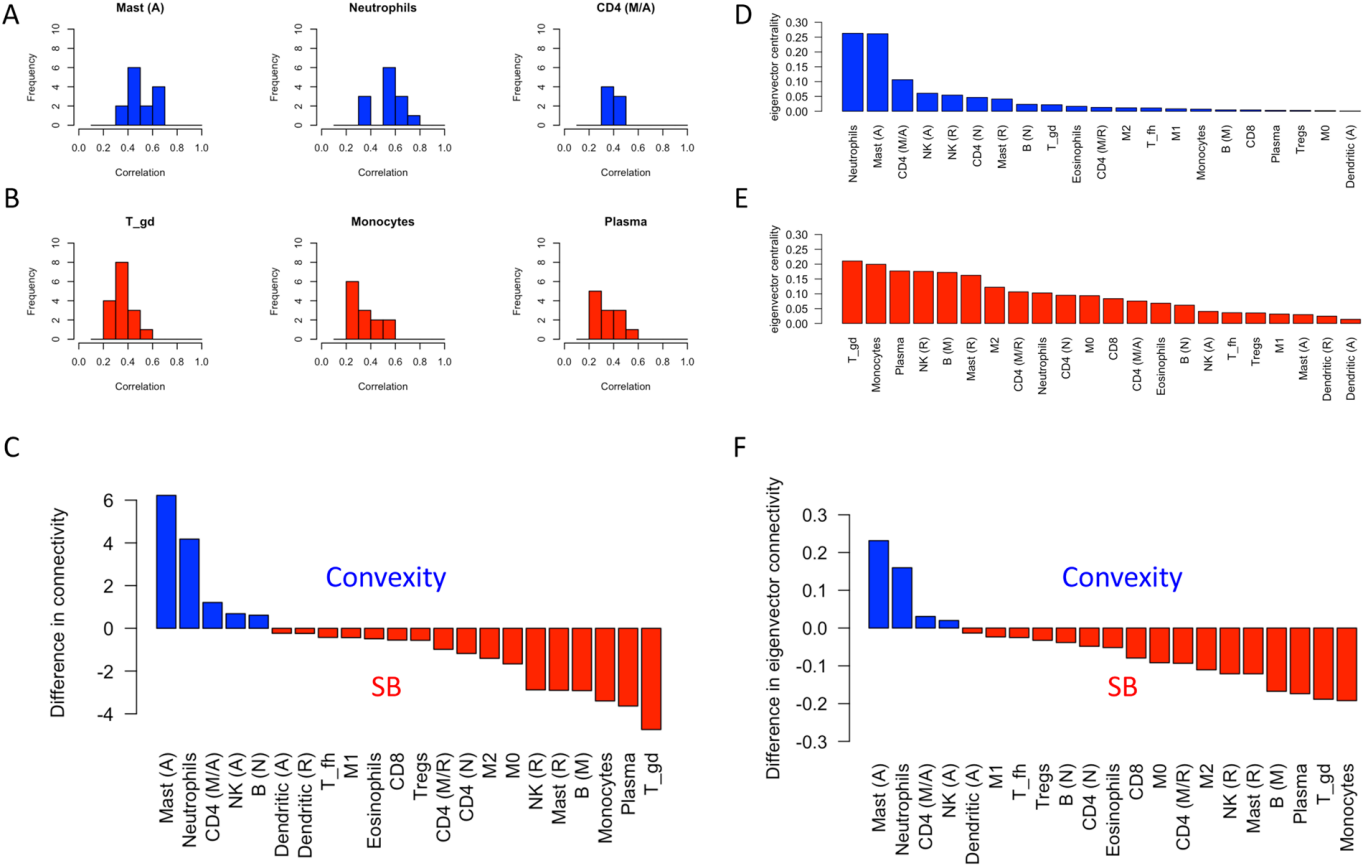
Immune cell connectivity of meningiomas by location. A-B: Histogram of Person’s correlations for the top 3 cell fractions, ranked by connectivity, of convexity (**A**) and skull base (**B**) meningiomas. C: Difference between cell connectivity values, comparing convexity to skull base (“SB”), where positive values (blue) indicate greater connectivity in convexity while negative values (red) indicate greater connectivity in skull base meningiomas. D and E: Ranking of eigenvector centrality of each cell type for convexity (**D**) and skull base (**E**) tumours. F: Difference between eigenvector centrality, with the same conventions as (**C**). Note the highly connected mast cells and neutrophils in convexity meningiomas and T gamma-delta cells, monocytes, and plasma cells in skull base (“SB”) meningiomas. A = activated, M = mature, N = naïve, fh = follicular helper, gd = gamma-delta, R = resting.

Given the relative complexity of these networks, we sought to analyze the relative “activity” of each immune cell by probing the distribution of their correlations with cytokine expression levels^19^. Analysis of cell “connectivity” (the area under this distribution) as well as eigenvector centrality (an established measure of node influence within a network) demonstrates the importance of mast cells and neutrophils in convexity meningiomas and of gamma-delta T cells, monocytes, and plasma cells in skull base meningiomas (Fig. 3).

## Discussion

We present a genetic meta-analysis correlating transcriptomics profile with meningioma location. Using a systems biology approach, we demonstrate an upregulation of various immune processes in skull base meningiomas compared to convexity (hemispheric) meningiomas. We further investigated the immune landscape of meningiomas by combining leukocyte cell fraction with cytokine expression profile in a network-based analysis. Gamma delta T cells were associated with the greatest cytokine connectivity and eigenvector centrality in our network of skull base meningiomas, while mast cells and neutrophils played the most central roles in the convexity network. This finding may explain the more benign biology of skull base meningiomas when compared to convexity meningiomas.

It is known that the more aggressive grade II and grade III meningiomas are less prevalent in the skull base compared to convexity meningiomas^7,20^. When comparing to meningiomas of the convexity using gene co-expression analysis, a key immune module showed significant upregulation in meningiomas of the skull base which was maintained after correcting for WHO grade. This suggests that cellular microenvironment, and in particular the immune microenvironment, may play an important role in modulating tumor behaviour and in explaining differences observed between the two subsets of meningioma being studied. The important role of tumour microenvironment has been well demonstrated in a variety of pan-cancer^12,21^ and central nervous system tumour studies^22^, and has been suggested in meningiomas^23–25^. Additionally, multiparameter flow cytometry studies have demonstrated a heterogenous composition of immune cells in meningiomas^23,25^. In our study, Gamma delta T cells were amongst the most active cell fraction in the skull base cohort based on both overall cytokine correlation and eigenvector centrality. Notably, this cell fraction is regarded as an inhibitor of tumor growth^26^. We also note importance of plasma cells and monocytes in this cohort, whose roles are less clear. Interestingly, tumour-infiltrating plasma cells have been associated with anti-tumoral activity in ovarian cancer^27^, and monocytes are noted to have both pro and anti-tumoral effects in cancer^28^. In convexity meningiomas, by contrast, we identified activated mast cells and neutrophils as the most connected fraction. Tumor-associated mast cells may support the oncogenic environment by releasing pro-tumorigenic stimulants^29^, thereby triggering angiogenesis, tumor cell proliferation/invasion, formation of lymphatic/blood vessel and facilitate the process of extravasation of cytokine-producing cells. Mast cells have been detected in upto 90% of high-grade meningioma tissue and has been also found to correlate with peritumoral edema^30^, a feature of aggressive biology in these tumours. Additionally, tumour-associated neutrophils have been implicated in cancer progression and poor clinical outcomes^31^.

Conventional analysis of transcriptomics data often relies on differential single-gene expression levels to filter out relevant patterns by comparing the “diseased” and “normal” tissue. These techniques may overlook relatively subtle effects across several highly-connected genes, which may nevertheless trigger a robust cellular mechanism. Gene expression networks are well suited for detecting small, additive biological signals that underlie relevant clinical phenotypes. In the first part of our analysis we used this well-established technique^14^ to show that immune processes correlate with location of meningiomas, and investigated this finding further using a network-based representation of the interactions between immune cell fractions and cytokine expression patterns. In this second technique, we used the sum of cell-cytokine correlations as a measure of immune cell “activity”, which is inspired by existing network based approaches^19^. Importantly, this output corresponds well to eigenvector centrality. Although further verification of this approach is needed, we highlight distinct cell fractions and interactions between skull base and convexity meningiomas, which align with known differences in clinical behaviour.

There are a number of limitations to this research which must be considered. Firstly, follow up data is lacking from all series we have includes which limits our ability to comment on tumour aggressiveness or recurrence rates, though our conclusions are nevertheless in alignment with prior knowledge on tumor immunology and the biology of convexity vs skull base meningiomas. We also do not have data on treatment regimens at the time of tissue collection, though as all patients undergo surgery for primary (non-recurrent) meningiomas and the vast majority of tumours our cohort are WHO grade 1, it’s very unlikely that any patients received neo-adjuvant treatment. Additionally, the proportion of grade II meningiomas is relatively low in our study (5.3%) compared to the prevalence in the population (20–30%). To address this, we have corrected for grade when correlating module gene expression with location. We also acknowledge that since skull base tumours are likely to produce symptoms and therefore be treated earlier, this may have an effect on the differences in observed biology. Notably, the case series of Magill *et al*.^7^ shows mean diameters of 2.6–3.6 cm in the skull base compared to 3.6–4.5 cm in convexity/falcine/parasaggital regions at the time of surgical excision. However, these findings are likely due to the well documented differences in genetic makeup^8,10^, biology, and growth rate of meningiomas. In non-skull base meningiomas, growth rate is higher and time to doubling is lower compared to skull base meningiomas^32^. Furthermore, the transformation of meningioma biology over time is quoted at a rate of 1–2%^33^ suggesting that the distinct biology is likely present from the initial occurrence of the meningioma rather than acquired over time. Consequently, the time of discovery/resection is unlikely to play a significant role. Finally, our assessment of cytokine activity is on the transcriptomics level as no secretory data was available, yet this approach still provides an indirect assessment of how “active” an immune cell type is in terms of cytokine synthesis. Additionally, we note that transcriptomics have been successfully used to infer autocrine and heterocrine cellular functions with good correlation between mRNA abundance and downstream protein synthesis. Such approaches have been successfully implemented to infer cell-cell interactions in lung development^34^ and aging of the mouse brain^35^. Importantly, we have been able to identify a robust, recurrent signal despite considerable data heterogeneity which lends itself to further confirmatory testing with biological assays.

## Conclusions

Our study is the first to computationally estimate immune cell fractions in location-specific meningioma tissue from bulk transcriptomics. We demonstrate distinct immune compositions between hemispheric and skull base meningiomas using a network-based approach that considers cell connectivity with cytokine transcriptomics. Gamma-delta T cells, with potentially tumour-suppressant activity, are more dominant in skull base meningiomas which is in keeping with a more benign biology. This is in contrast to the more central role of potentially oncogenic mast cells and neutrophils in convexity meningiomas. These findings give further insight into the immune microenvironment of meningiomas and may have implications on future strategies of immune modulation for this challenging disease.

## Methods

### Data preparation

All data was collected from the Gene Expression Omnibus (GEO), a public repository of high-throughput functional genomic data sets^36^. We used studies containing details on meningioma location (skull base and convexity) and WHO grade with corresponding gene expression data^11,37^, providing us with a cumulative sample size of 107 meningiomas (Table 1). All studies obtained microarray data from surgically excised primary meningioma tissue. Summary statistics for each study are presented in Table 1. We note that a diagram outlining the workflow can be found in Supplemental Fig. 1.

**Table 1:** Table summary of study population.

Sample ID	N patients (SB)	Mean Age (SD)	Sex (M:F)	WHO grade 1 2 3		
GSE88720^37^	12 (7)	56.2 (15.8)	4:7	10	2	0
GSE84263^11^	84 (53)	56.9 (12.3)	10:2	84	0	0
GSE77259^38^	11 (11)	52.9 (11.0)	66:18	7	4	0
Total	107 (71)	56.4 (12.6)	80:27	101	6	0

### Pre-processing of transcriptomics data

For each study, the microarray data was backgrounded corrected, quantile normalized, and log-2 transformed using the *Affy*^39^ and *Limma*^40^ R packages for Affymetrix and Illumina platforms, respectively. After removing genes that were not common across these studies (such that the cohorts could be merged into a single matrix) we were left with 19,011 genes. The 3 studies were then merged, scaled to a global mean and standard deviation of 0 and 1, respectively^41^, and batch-corrected using *ComBat*, a well-established empirical Bayes approach^42^. The resultant data matrix was used during all subsequent analysis.

### Co-expression network analysis

We performed WGCNA of gene expression data using R (version 3.5.1) to construct a co-expression network and identify biological modules which map to meningioma location. Pairwise gene correlations were soft-thresholded with an exponent of 20 to approximate scale-free topology, which was ultimately transformed into a biologically-inspired “Topological Overlap Matrix” (TOM), which measures pairwise gene similarity in terms of shared topology within the full network^14^. Highly similar genes are then grouped into an adaptive hierarchical clustering tree (dendrogram), yielding “modules” with a minimum size of 30 genes. The gene expression profile of each module is represented by a meta-gene computed as its first principal component, an established method. The meta-gene score for each module is then compared between skull base and convexity meningiomas, with significance considered at Mann Whitney p < 0.05. As an exploration of the relationship between cytokine activity and module expression, we label cytokines whose expression levels are significantly positively associated with module meta-gene expression (Pearson correlation>0.6, p < 0.05). Finally, to assess for network robustness, we select the 10 most correlated genes to the module eigengene from all modules which are significantly associated with tumour location and use their expression as inputs into a logistic regression model to predict location.

### Module-based qualitative analysis

Genes in each of the identified modules were annotated by the Database for Annotation, Visualization and Integrated Discovery (DAVID version 6.8)^43^.

### Deconvolution of tumor bulk expression signal

We used CIBERSORT^44^ (Package *EpiDISH* version 1.4.1), a well-established technique to estimate immune cell fractions from our gene expression data. Briefly, the method uses support vector regression to compare bulk transcriptomic data from previously derived signature matrices from 22 different immune cells in order to estimate the relative prevalence of each of these 22 immune cells in the bulk data. The specifics of this technique are beyond the scope of this paper and are described in detail in the attached reference. Notably, the input gene expression data is not log transformed and is scaled to a global mean and standard deviation of 0 and 1, respectively.

### Network analysis of immune cell fractions and expression of cytokines

The complex relationships between immune cells and cytokine expression levels were visualized using network analysis, which demonstrates associations that are otherwise difficult to appreciate. Such network-based approaches have revolutionized research into phenom-genotype similarities and elucidated the genetic basis of diseases^45,46^, predicted pathways of disease progression^19^, and identified novel drug targets^47^. Nodes represent immune cells (CIBERSORT output) and list of 35 known cytokines derived from the literature^21,48^ (Supplemental data). Edges represent Pearson correlations, wherein a significance cut-off of <0.05 and effect size cutoff of>0.6 are used. Node size is proportional to the eigenvector centrality as computed by the CYTOSCAPE (version 3.6.1) interface. We also compute an unweighted eigenvector centrality score (a measure of node influence within a network which is independent of Pearson correlations) for each cell types using a network wherein adjacency is defined by significant correlations (p < 0.05) in order to assess the degree of influence of each cell within each network. We also define “connectivity” as the biological activity of each immune cell (i.e. its overall expression of cytokines), computed as the sum of all significant (p < 0.05) Pearson correlations between a cell type and the expression levels of 35 pre-determined cytokines (Supplementary data). Cell types were ranked based on both of these metrics.

## Supplementary information

Supplemental figures.

Supplementary Dataset 1.

Supplementary Dataset 2.

Supplementary Dataset 3.

Supplementary Dataset 4.

Supplementary Dataset 7.

Supplementary Dataset 5.

Supplementary Dataset 6.

R codes.

## Data Availability

All data referenced in this study is publicly available through the open repository Gene Expression Omnibus (https://www.ncbi.nlm.nih.gov/geo/).
