# Supplementary material for: Landscape of immune cell gene expression is unique in predominantly WHO grade 1 skull base meningiomas when compared to convexity
Source: Sci Rep. 2020 Jun 3;10:9065. doi: 10.1038/s41598-020-65365-7 (PMC7270140; doi:10.1038/s41598-020-65365-7)
Supplement: Supplementary file 1 — Supplemental figures. [file 41598_2020_65365_MOESM1_ESM.pdf]

SUPPLEMENTAL FIGURES

"Landscape of immune cell gene expression is unique in predominantly WHO grade 1 skull base meningiomas when compared to convexity"

Zsolt Zador[1], Alexander P. Landry[1], Michael Balas[1], Michael D. Cusimano[1]

1) Division of Neurosurgery, Department of Surgery, St. Michael's Hospital, Toronto, ON, Canada

Corresponding Author:

Zsolt Zador

Division of Neurosurgery, Department of Surgery, St. Michael's Hospital,

Email: zadzso@gmail.com

Phone: 00-416-864-5312

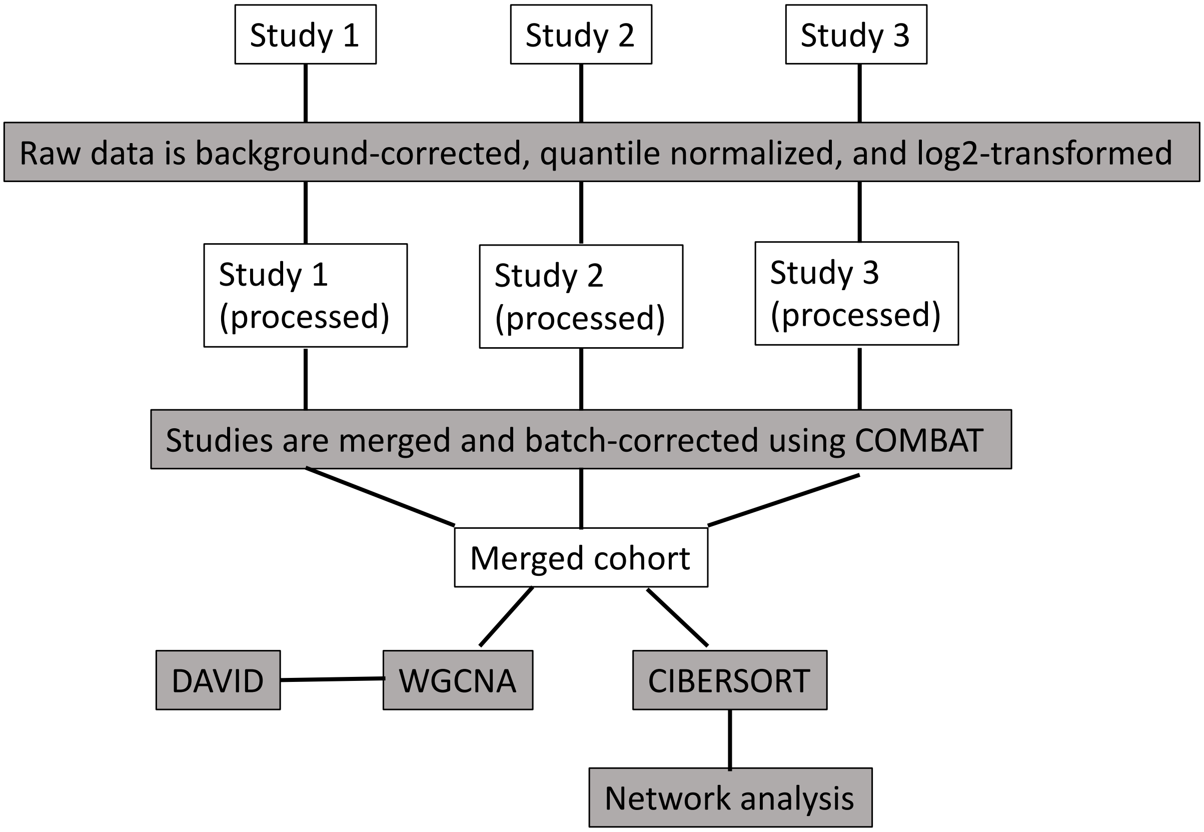

Supplemental Figure 1: Overview of this study's workflow.

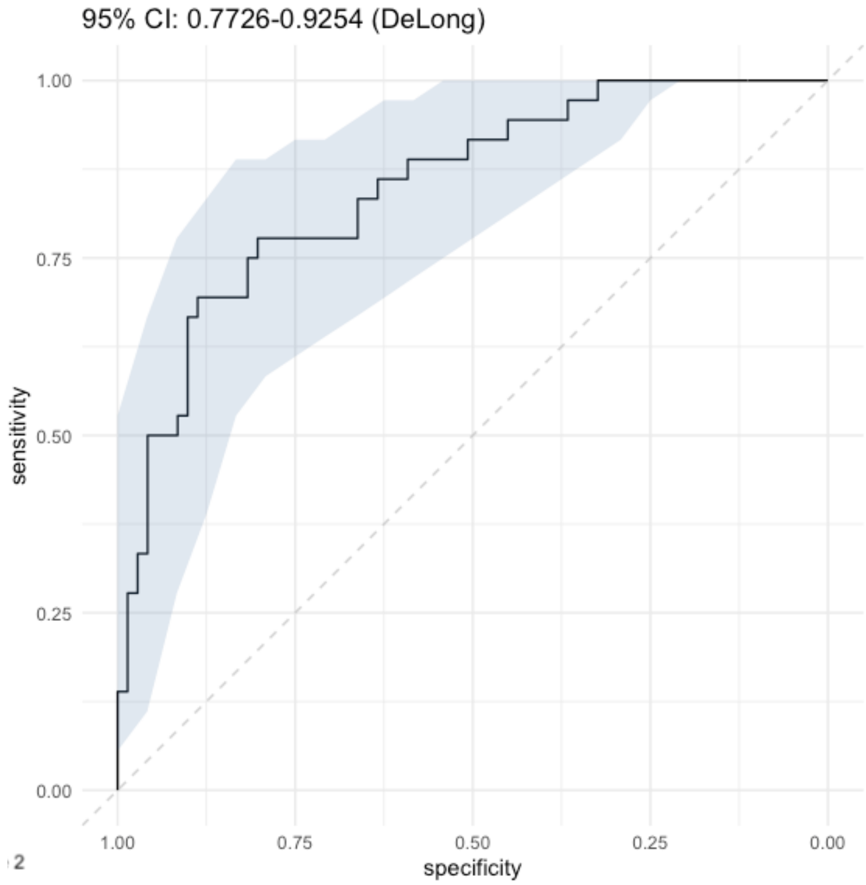

	Actual	
**Predicted**	Skull Base	Convexity
Skull Base	64	14
Convexity	7	22

Supplemental Figure 2: Receiver-operating characteristic curve of top 10 genes by correlation to module eigengene of 3 modules which correlate significantly to location, with attached confusion matrix.

Cytokine List:

CCL2, CCL3, CCL4, CXCL1, CXCL8, IFNG, IL10, IL12A, IL12B, IL1B, IL1R2, IL1RN, IL4, IL6, IL6R, TNF, TNFRSF1A, TNFRSF1B, BTK, CD244, CD274, CSF1, CSF2, EGF, GZMB, HAVCR2, IFNG, IL13, IL1A, LAG3, MMP9, PDCD1, PLAU, THBD, VEGFA
