# Supplementary material for: Landscape of immune cell gene expression is unique in predominantly WHO grade 1 skull base meningiomas when compared to convexity
Source: Sci Rep. 2020 Jun 3;10:9065. doi: 10.1038/s41598-020-65365-7 (PMC7270140; doi:10.1038/s41598-020-65365-7)
Supplement: Supplementary file 9 — R codes. [file 41598_2020_65365_MOESM9_ESM.docx]

**SUPPLEMENTAL R CODES**

**“Landscape of immune cell gene expression is unique in predominantly WHO grade 1 skull base meningiomas when compared to convexity”**

Zsolt Zador^1^, Alexander P. Landry^1^, Michael Balas^1^, Michael D. Cusimano^1^

1. Division of Neurosurgery, Department of Surgery, St. Michael’s Hospital, Toronto, ON, Canada

**Corresponding Author:**

Zsolt Zador

Division of Neurosurgery, Department of Surgery, St. Michael’s Hospital,

Email: zadzso@gmail.com

Phone: 00-416-864-5312

**#Gene normalization**

#Load the necessary libraries

library(GEOquery)

library(affy)

library(gcrma)

library(hugene10stv1cdf)

library(hugene10stv1probe)

library(hugene10stprobeset.db)

library(hugene10sttranscriptcluster.db)

library(hugene10stcdf)

library(hgu133plus2.db)

library(hgu133plus2cdf)

library(hgu133plus2probe)

library(hgu133aprobe)

library(hgu133a.db)

library(hgu133acdf)

library(oligo)

library(pd.hugene.2.1.st)

library(WGCNA)

library(hugene10sttranscriptcluster.db)

library(hugene21sttranscriptcluster.db)

library(hgu133plus2.db)

library(illuminaHumanv4.db)

library(AnnotationDbi)

library(limma)

library(illuminaio)

annotateProbes = function(platform) {

symbol = AnnotationDbi::select(platform, keys=keys(platform), columns = c("SYMBOL"))

symbol = na.omit(symbol)

symbol = symbol[!duplicated(symbol[,1]), ]

return(symbol)

}

gse = "GSE77259" #GSE88720 (AFFY), GSE84263 (ILLUMINA), GSE77259 (AFFY)

# Create new directory for GSE data

dir.create(file.path(getwd(), gse))

# Access the GEO Series data

getGeo = getGEO(gse, GSEMatrix = TRUE, getGPL=FALSE, destdir = gse);

# Access the raw CEL file data in the current base directory

getGEOSuppFiles(gse, makeDirectory = FALSE, baseDir = gse)

#### ILLUMINA

# doesn't work, do manually (uncompress file and put in folder called TXT_data)

#untar(paste(gse, "/", gse, "_Non-normalized_data.txt.gz", sep=""), exdir=paste(gse, "/TXT_data", sep=""))

txts = list.files(paste(gse, "/TXT_data", sep=""), pattern="txt", ignore.case=TRUE, full.names=TRUE)

data = read.ilmn(txts, probeid="ID_REF", expr="Signal")

# Keep subjects that meet criteria

subjects = read.csv('GSE84263_Phenotype.tsv', sep='\t')

data = data[ , intersect(subjects$ExpressionID, colnames(data))]

data = data[, !colnames(data) %in% c("7911739001_F", "9664921051_A")] # Get rid of Post fossa tumours

# neqc is a between array normalization function customized for Illumina BeadChips. Several other methods within the Limma package

data.norm = neqc(data)

data = data.norm$E

# Annotate probes to genes

probeList = annotateProbes(illuminaHumanv4.db)[,1]

geneList = annotateProbes(illuminaHumanv4.db)[,2]

data = (collapseRows(as.data.frame(data), geneList, probeList))[[1]]

gse84263_genes = data

# Save as R data object

save(gse84263_genes, file=paste(gse, "_genes.rda", sep=""))

### AFFY

# Unpack the CEL files

untar(paste(gse, "/", gse, "_RAW.tar", sep=""), exdir=paste(gse, "/CEL_DATA", sep=""))

cels = list.files(paste(gse, "/CEL_data", sep=""), pattern="cel.gz", ignore.case=TRUE, full.names=TRUE)

# DELETE FILES WHICH WON'T BE USED

if (gse=="GSE88720") {

x = c("GSM2344707", "GSM2344697","GSM2344696") # Remove controls and post fossa tumours

#x = "GSM2344707" # Just control

} else if (gse=="GSE77259") {

x = c("GSM2046764","GSM2046757", "GSM2046763") # Remove spine tumour and post fossa tumours

#x = "GSM2046764" # Just spinal tumour

}

# Remove files specified in 'x' from cels list

cels = cels[!cels %in% grep(paste0(x, collapse = "|"), cels, value = T)]

# import CEL files containing raw probe-level data into an R AffyBatch object

data = read.celfiles(cels)

data.rma = rma(data)

# Get the expression estimates for each array

rma = exprs(data.rma)

# Annotate probes to genes

if (gse=="GSE88720") {

probeList = annotateProbes(hugene21sttranscriptcluster.db)[,1]

geneList = annotateProbes(hugene21sttranscriptcluster.db)[,2]

rma = (collapseRows(as.data.frame(rma), geneList, probeList))[[1]]

} else if (gse=="GSE77259") {

probeList = annotateProbes(hugene10sttranscriptcluster.db)[,1]

geneList = annotateProbes(hugene10sttranscriptcluster.db)[,2]

rma = (collapseRows(as.data.frame(rma), geneList, probeList))[[1]]

}

# Clean colnames

colnames(rma) = gsub("\\_.*","",colnames(rma))

if (gse=="GSE88720") {

gse88720_genes = rma

# Save as R data object

save(gse88720_genes, file=paste(gse, "_genes.rda", sep=""))

} else if (gse=="GSE77259") {

gse77259_genes = rma

# Save as R data object

save(gse77259_genes, file=paste(gse, "_genes.rda", sep=""))

}

**#COMBAT**

#location meningoma

load("GSE88720_genes.rda")

load("GSE84263_genes.rda")

load("GSE77259_genes.rda")

commonGenes = intersect(rownames(gse88720_genes), rownames(gse84263_genes))

commonGenes = intersect(commonGenes, rownames(gse77259_genes))

gse88720_genes = gse88720_genes[commonGenes, ]

gse84263_genes = gse84263_genes[commonGenes, ]

gse77259_genes = gse77259_genes[commonGenes, ]

library(sva)

library(COMBAT)

library(Biobase)

library(bladderbatch)

library(snpStats)

library(matrixStats)

#GSE77259-hugene10stv1,

expr1 <- gse77259_genes - rowMeans(gse77259_genes)

expr1 <- t(t(expr1) - colMeans(expr1))

e1 <- expr1/rowSds(expr1)

e1 <- t(t(e1)/colSds(e1))

batch1 = rep(1,ncol(gse77259_genes))

#GSE88720-hugene21st,

expr2 <- gse88720_genes - rowMeans(gse88720_genes)

expr2 <- t(t(expr2) - colMeans(expr2))

e2 <- expr2/rowSds(expr2)

e2 <- t(t(e2)/colSds(e2))

batch2 = rep(2,ncol(gse88720_genes))

#illumina GSE84263 86

expr3 <- gse84263_genes - rowMeans(gse84263_genes)

expr3 <- t(t(expr3) - colMeans(expr3))

e3 <- expr3/rowSds(expr3)

e3 <- t(t(e3)/colSds(e3))

# 3 Batches

#batch3 = rep(3,ncol(gse84263_genes))

# 8 Batches

datTraits1 <- read.csv("~/Documents/KRSS/Meningioma/GSE84263/supplementary.tsv", sep="\t")

datTraits1 = subset(datTraits1, datTraits1$ExpressionID %in% colnames(gse84263_genes))

batch3 <- as.numeric(datTraits1$Batch) #this dataset has 86 samples and 8 batches

batch3[which(batch3 == 1)] = 7

batch3[which(batch3 == 2)] = 8

#prepare data for ComBat

expr <- cbind(e1,e2,e3)

batch = c(batch1, batch2, batch3)

#run ComBat

edata <- ComBat(dat = expr, batch = batch, mod=NULL, par.prior = TRUE, prior.plots = FALSE)

save(edata, batch, file = "edata_batch.rda") #object edata

**#CIBERSORT**

setwd("/Users/alexlandry/Documents/Research/Meningioma/Location")

library("EpiDISH")

library("sva")

library("matrixStats")

load("datTraits.rda")

load("edata_batch_new_Michael.rda")

input2 = edata

gexpr1 = as.matrix(2^(input2))

expr <- gexpr1 - rowMeans(gexpr1)

expr <- t(t(expr) - colMeans(expr))

e <- expr/rowSds(expr)

e <- t(t(e)/colSds(e))

gexpr = e

ref.m1 = read.csv("LM22.csv", row.names = 1)

intersect = intersect(rownames(ref.m1),rownames(gexpr))

ref.gbm = ref.m1[intersect,]

ref.gbm <- ref.gbm - rowMeans(ref.gbm)

ref.gbm <- t(t(ref.gbm) - colMeans(ref.gbm))

ref.gbm <- ref.gbm/rowSds(ref.gbm)

ref.gbm <- t(t(ref.gbm)/colSds(ref.gbm))

gbmout <- epidish(gexpr, as.matrix(ref.gbm), method = "CBS", nu.v = c(0.25,0.5,0.75))

colnames(gbmout$estF) = c("B (N)","B (M)","Plasma","CD8","CD4 (N)","CD4 (M/R)","CD4 (M/A)","T_fh","Tregs","T_gd","NK (R)","NK (A)","Monocytes","M0","M1","M2","Dendritic (R)","Dendritic (A)","Mast (R)","Mast (A)","Eosinophils","Neutrophils")

**#WGCNA**

setwd("/Users/alexlandry/Documents/Research/Meningioma/Location")

load("edata_batch_new_Michael.rda")

load("datTraits.rda")

library(WGCNA)

library(flashClust)

options(stringsAsFactors = FALSE);

allowWGCNAThreads()

# Choose a set of soft-thresholding powers

powers = c(c(1:10), seq(from = 12, to=30, by=2))

# Call the network topology analysis function

sft = pickSoftThreshold(t(edata), powerVector = powers, verbose = 5, RsquaredCut = 0.9)

# Plot the results:

sizeGrWindow(9, 5)

par(mfrow = c(1,2));

cex1 = 0.9;

# Scale-free topology fit index as a function of the soft-thresholding power

plot(sft$fitIndices[,1], -sign(sft$fitIndices[,3])*sft$fitIndices[,2],

xlab="Soft Threshold (power)",ylab="Scale Free Topology Model Fit,signed R^2",type="n",

main = paste("Scale independence"));

text(sft$fitIndices[,1], -sign(sft$fitIndices[,3])*sft$fitIndices[,2],

labels=powers,cex=cex1,col="red");

# this line corresponds to using an R^2 cut-off of h

abline(h=0.90,col="red")

#SET SOFTPOWER AS LOWEST INTEGER WHICH ACHIEVES R^2 >0.9 (above red line)

#building TOM

softPower = sft$powerEstimate;

adjacencyB1 = adjacency(t(edata),power=softPower,type="signed");

diag(adjacencyB1)=0

dissTOMB1 = 1-TOMsimilarity(adjacencyB1, TOMType="signed")

geneTreeB1 = flashClust(as.dist(dissTOMB1), method="average")

#tree cut - module detection

tree = cutreeHybrid(dendro = geneTreeB1, pamStage=FALSE,

minClusterSize = 30, cutHeight = 0.99,

deepSplit = 0, distM = dissTOMB1)

modulesB1=labels2colors(tree$labels);

plotDendroAndColors(geneTreeB1, modulesB1, main = "",dendroLabels=FALSE);

#meta genes

PCs1A = moduleEigengenes(t(edata), colors=modulesB1)

ME_1A = PCs1A$eigengenes

rownames(ME_1A) <- colnames(edata)

distPC1A = 1-abs(cor(ME_1A,use="p"))

distPC1A = ifelse(is.na(distPC1A), 0, distPC1A)

pcTree1A = hclust(as.dist(distPC1A),method="a")

MDS_1A = cmdscale(as.dist(distPC1A),2)

colorsB1 = names(table(modulesB1))

nGenes = nrow(edata);

Gene = rownames(edata)

geneModuleMembership = signedKME(t(edata), ME_1A)

colnames(geneModuleMembership)=paste("PC",colorsB1,".cor",sep="");

topGenesKME = NULL

for (c in 1:length(colorsB1)){

kMErank1 = rank(-geneModuleMembership[,c])

topGenesKME = cbind(topGenesKME,Gene[kMErank1<=10])

}; colnames(topGenesKME) = colorsB1

topGenesKME

names(modulesB1) <- rownames(edata)

names(modulesB1) <- rownames(geneModuleMembership)

#Make list containing all module genes, rnked by kME

genes = vector(mode="list", length = length(colorsB1));

for (c in 1:length(colorsB1)){

z <- names(modulesB1[modulesB1 == colorsB1[c]])

q <- match(z, rownames(geneModuleMembership))

gmm <- geneModuleMembership[q,c]

genes[[c]] <- cbind(z, rank(-gmm), gmm)

};

names(genes) = colorsB1

#use this if need to transfer to DAVID via copy/paste

print(as.data.frame(genes[["blue"]][,1]),quote = FALSE,row.names = FALSE)

print(as.data.frame(genes[[13]][,1]), quote = FALSE, row.names = FALSE)

#save data

save(modulesB1,ME_1A,colorsB1,genes, file = "WGCNA_output_30_gene_minimum.rda")

#finding specific genes in modules (if wanted)

a = vector(mode = "list", length = length(genes))

for (i in 1:length(genes)){

a[i] = genes[[i]][genes[[i]][,1] == "SMO"]

}

#find modules significantly associated with location

p= NULL

for (j in 1:ncol(ME_1A)){

p[j] <- wilcox.test(as.numeric(ME_1A[which(datTraits$loc == 1),j]),as.numeric(ME_1A[which(datTraits$loc == 2),j]))$p.value

}

**#Module correlation with location labels**

library(WGCNA)

library(flashClust)

library(Rtsne)

library("EpiDISH")

library("sva")

### ~~~ ######################################################################## ~~~ ###

### ~~~ Statistical Significance of Phenotype Correlation with Module Eigengenes ~~~ ###

### ~~~ ######################################################################## ~~~ ###

p = NULL

colours = names(table(moduleColours))

skull_base = MEs[which(datTraits$loc == 1), ]

convexity = MEs[which(datTraits$loc == 2), ]

data_cor = cbind(MEs, datTraits)

logistic_p = matrix(nrow = length(colours), ncol = 2)

for (i in 1:length(colours)){

data = c(g1=skull_base[i], g2=convexity[i])

p[i] = kruskal.test(data)$p.value

model = glm(factor(loc) ~ MEs[,i] + who + age + sex, data = data_cor, family = binomial)

p_ME = summary(model)$coefficients[2,4]

p_grade = summary(model)$coefficients[3,4]

logistic_p[i, ] = cbind(p_ME, p_grade)

}

min(p) #0.09143672

colours[(which.min(p))] # turquoise - 0.004600378 / 0.00463722

colnames(logistic_p) = c("p_ME", "p_Grade")

rownames(logistic_p) = colours

print("meningioma ME significance values while accounting for WHO grade:")

print(logistic_p)

# Boxplot to visualize data

# Choose most significant module (i.e. lowest p-value):

module_pValue = which.min(p)

module_pValue = 13

sizeGrWindow(12, 9)

verboseBoxplot(as.numeric(MEs[,module_pValue]), datTraits$loc, main=paste(colours[module_pValue], 'module:'),

las=1, xlab="Location (1=Skull Base, 2=Convexity)",

ylab="", notch = FALSE, varWidth = TRUE, addScatterplot = FALSE, KruskalTest = TRUE)

### ~~~ ###################################### ~~~ ###

### ~~~ Predictive Power of Top N Module Genes ~~~ ###

### ~~~ ###################################### ~~~ ###

top_n_genes = 10

datKME=signedKME(t(edata), MEs, outputColumnName="MM.")

gene_expr_vals = as.data.frame(t(edata))[moduleColours=='turquoise']

turqKME = datKME[order(datKME[,13], decreasing = TRUE),][,13, drop=FALSE]

turqKME = subset(turqKME, rownames(turqKME) %in% colnames(gene_expr_vals))

top_turq = head(rownames(turqKME), top_n_genes)

gene_expr_vals = as.data.frame(t(edata))[moduleColours=='greenyellow']

gyKME = datKME[order(datKME[,5], decreasing = TRUE),][,5, drop=FALSE]

gyKME = subset(gyKME, rownames(gyKME) %in% colnames(gene_expr_vals))

top_gy = head(rownames(gyKME), top_n_genes)

gene_expr_vals = as.data.frame(t(edata))[moduleColours=='purple']

purpKME = datKME[order(datKME[,9], decreasing = TRUE),][,9, drop=FALSE]

purpKME = subset(purpKME, rownames(purpKME) %in% colnames(gene_expr_vals))

top_purp = head(rownames(purpKME), top_n_genes)

gene_expr_vals = as.data.frame(t(edata))[moduleColours=='turquoise']

gene_expr_vals = cbind(gene_expr_vals, as.data.frame(t(edata))[moduleColours=='greenyellow'])

gene_expr_vals = cbind(gene_expr_vals, as.data.frame(t(edata))[moduleColours=='purple'])

top_genes = union(union(top_turq, top_gy), top_purp)

gene_expr_vals = gene_expr_vals[,colnames(gene_expr_vals)%in%top_genes]

gene_expr_vals$loc = datTraits$loc

library(caret)

# define training control

train_control = trainControl(method = "repeatedcv", number = 3, repeats = 3)

# train the model on training set

model <- train(factor(loc) ~ .,

data = gene_expr_vals,

trControl = train_control,

method = "glm",

family=binomial(link = "logit"))

pred = predict(model, newdata=gene_expr_vals)

confusionMatrix(data=pred, as.factor(gene_expr_vals$loc))

ROC = roc(factor(datTraits$loc) ~ model$finalModel$fitted.values, main='Predictive Power of Top 30 Genes from Significant Modules')

plot(ROC)

auc(ROC)

### ~~~ ###################################### ~~~ ###

### ~~~ Export Module Gene Data for Annotation ~~~ ###

### ~~~ ###################################### ~~~ ###

# Export most significant module

genes = names(as.data.frame(t(edata))[moduleColours==module])

filename = paste(paste("gene_ontology/", location_name, sep=""), "_loc.csv", sep="")

write.table(as.data.frame(genes), file=filename,

row.names = FALSE, col.names = FALSE, quote = FALSE)

# Export all modules

for (colour in colours){

genes = names(as.data.frame(t(edata))[moduleColours==colour])

filename = paste("gene_ontology/", colour, "_module.csv", sep="")

write.table(as.data.frame(genes), file=filename,

row.names = FALSE, col.names = FALSE, quote = FALSE)

}

# For Modules with more than 3,000 genes (limit of DAVID)

turq = as.data.frame(t(edata))[moduleColours == "turquoise"]

temp = as.data.frame(cor(turq, MEs$MEturquoise, use="p"))

topTurq = temp[order(-temp$V1), , drop = FALSE]

topTurq = head(topTurq, 3000)

topTurq = unlist(t(topTurq))

topTurq = rownames(t(topTurq))

write.table(as.data.frame(topTurq), file="gene_ontology/turquoise_module.csv",

row.names = FALSE, col.names = FALSE, quote = FALSE)

### ~~~ ###################################### ~~~ ###

### ~~~ ###################################### ~~~ ###

**#Network analysis**

setwd("/Users/alexlandry/Documents/Research/Meningioma/Location")

load("cytokines35.rda")

load("gbmout_new.rda")

load("edata_batch_new_Michael.rda")

load("datTraits.rda")

citokines = edata[rownames(edata) %in% cytokines35,]

fractions = gbmout$estF[colnames(edata),]

labels = datTraits$loc

#save(labels,citokines, fractions, file = "for_network_connectivity.rda")

#LOAD IN DATA HERE

cyt = as.data.frame(t(citokines))

input_data = cbind(cyt, fractions)

try = input_data

try1 = try[which(labels == 1),]

try2 = try[which(labels == 2),]

#USE LINE BELOW TO CLEAN LOCATION DATA

#NORMALIZE each column of data to range 0-1 (if using continuous variables-not 100% necessary I don't think)

#try = input_data

#for (j in 1:ncol(try)){

# try[,j] = (try[,j]-min(try[,j], na.rm = TRUE))/max((try[,j]-min(try[,j], na.rm = TRUE)),na.rm = TRUE)

#}

#=======NETWORK ANALYSIS==================================================

try = try1

#Initialize matrices (co = pearson correlation; pco = associated p-value)

co = matrix(nrow = ncol(try), ncol = ncol(try))

pco = matrix(nrow = ncol(try), ncol = ncol(try))

for (l in 1:ncol(try)){

for (m in 1:ncol(try)){

if (l == m){

co[l,m] = 0

pco[l,m] = 1

} else {

co[l,m] = cor(try[,l],try[,m], use = "complete.obs")

pco[l,m] = cor.test(try[,l],try[,m], use = "complete.obs")$p.value

}

}

}

#Permutations of names combinations (name 1 and name2)

names1 = rep(colnames(try), nrow(co))

names2 = NULL

for (k in 1:nrow(co)){

names2 = c(names2,rep(colnames(try)[k],nrow(co)))

}

#make matrix with 4 columns: name1, name2, pearson coefficient, p-value. Correlation is between name1 and name2

tryagain = cbind(names1,names2,as.vector(co), as.vector(pco))

#remove repeats

tryagain2 = tryagain[!duplicated(apply(tryagain,1,function(x) paste(sort(x),collapse=''))),]

#make data frame, take absolute value of correlation

tryagain2 = as.data.frame(tryagain2)

tryagain2$names1 = as.character(tryagain2$names1)

tryagain2$names2 = as.character(tryagain2$names2)

tryagain2$V3 = abs(as.numeric(as.character(tryagain2$V3)))

tryagain2$V4 = as.numeric(as.character(tryagain2$V4))

#filter (if you want). Here I use a cutoff of p < 0.05 and abs(cor) > 0.4

tryagain2 = tryagain2[which(tryagain2[,4] < 0.05),]

tryagain2 = tryagain2[which(abs(tryagain2[,3]) > 0.6),]

#write this 4 column file as a .csv file. Can go directly into Cytoscape

write.csv(tryagain2, file = "network_sb_convexity_p0.05.csv")

#Below matrices derived from cytoscape

eigen_con = read.table("network_35_cytokines/Final/eigenvector_convexity_allp0.05.txt", row.names = 1)

eigen_con = eigen_con[-c(1,2),-2]

eigenc = eigen_con$V4

eigenc = as.numeric(eigenc)

names(eigenc) = eigen_con$V2

eigen_sb = read.table("network_35_cytokines/Final/eigenvector_sb_allp0.05.txt", row.names = 1)

eigen_sb = eigen_sb[-c(1,2),-2]

eigens = eigen_sb$V4

eigens = as.numeric(eigens)

names(eigens) = eigen_sb$V2

par(mfrow = c(2,1))

par(mar = c(6,6,3,3))

barplot(rev(sort(eigenc[colnames(gbmout$estF)])), las = 2, col = "blue", ylim = c(0,0.3), ylab = "eigenvector centrality")

barplot(rev(sort(eigens[colnames(gbmout$estF)])), las = 2, col = "red", ylim = c(0,0.3), ylab = "eigenvector centrality")

barplot(rev(sort(eigenc[colnames(gbmout$estF)] - eigens[colnames(gbmout$estF)])), las = 2, ylim = c(-0.3,0.3), col = c(rep("blue",4),rep("red",18)), ylab = "Difference in eigenvector connectivity")

**#Cell fraction and cytokine network analysis**

setwd("/Users/alexlandry/Documents/Research/Meningioma/Location")

load("cytokines35.rda")

#load("gbmout_new.rda")

load("datTraits.rda")

load("edata_batch_new_Michael.rda")

cyt = edata[rownames(edata) %in% cytokines35,]

call = as.data.frame(t(cyt))

fractions = gbmout$estF

#location

c = call[which(datTraits$loc == 1),]

fractions = fractions[rownames(c),]

cor = matrix(nrow = ncol(fractions), ncol = ncol(c))

pcor = matrix(nrow = ncol(fractions), ncol = ncol(c))

for (i in 1:ncol(fractions)){

for (j in 1:ncol(c)){

cor[i,j] = cor(fractions[,i],c[,j])

pcor[i,j] = cor.test(fractions[,i],c[,j])$p.value

}

}

rownames(cor) = colnames(fractions)

colnames(cor) = colnames(c)

rownames(pcor) = colnames(fractions)

colnames(pcor) = colnames(c)

cor = abs(cor)

connectivity = vector()

for (k in 1:22){

connectivity[k] = sum(cor[k,which(pcor[k,]<0.05)])

}

names(connectivity) = rownames(cor)

index = rev(order(connectivity))[1:5]

cor1 = cor

pcor1 = pcor

index1 = index

connectivity1 = connectivity

#=====

fractions = gbmout$estF

c = call[which(datTraits$loc == 2),]

fractions = fractions[rownames(c),]

cor = matrix(nrow = ncol(fractions), ncol = ncol(c))

pcor = matrix(nrow = ncol(fractions), ncol = ncol(c))

for (i in 1:ncol(fractions)){

for (j in 1:ncol(c)){

cor[i,j] = cor(fractions[,i],c[,j])

pcor[i,j] = cor.test(fractions[,i],c[,j])$p.value

}

}

rownames(cor) = colnames(fractions)

colnames(cor) = colnames(c)

rownames(pcor) = colnames(fractions)

colnames(pcor) = colnames(c)

cor = abs(cor)

connectivity = vector()

for (k in 1:22){

connectivity[k] = sum(cor[k,which(pcor[k,]<0.05)])

}

names(connectivity) = rownames(cor)

index = rev(order(connectivity))[1:5]

cor2 = cor

pcor2 = pcor

index2 = index

connectivity2 = connectivity

par(mfrow = c(2,5))

for (a in 1:length(index2)){

hist(cor2[index2[a],which(pcor2[index2[a],]<0.05)], xlim = c(0,1), ylim = c(0,10), main = rownames(cor2)[index2[a]], breaks = c(1:10)/10, xlab = "Correlation", col ="blue")

}

for (a in 1:length(index1)){

hist(cor1[index1[a],which(pcor1[index1[a],]<0.05)], xlim = c(0,1), ylim = c(0,10), main = rownames(cor1)[index1[a]], breaks = c(1:10)/10, xlab = "Correlation", col = "red")

}

connectivity = rbind(connectivity1,connectivity2)

rownames(connectivity) = c("SB","Convexity")

heatmap.plus(connectivity, Rowv = NA)

barplot(rev(sort(connectivity2 - connectivity1)), las =2, ylab = "Difference in connectivity", col = c(rep("blue",5),rep("red",17)))

#library(heatmap.plus)

#heatmap.plus(cor, Rowv = NA, cexRow = 1)

**#Module cytokine correlation**

cor = matrix(nrow = length(cytokines35), ncol = ncol(ME_1A))

pcor = matrix(nrow = length(cytokines35), ncol = ncol(ME_1A))

for (j in 1:ncol(ME_1A)){

for (x in 1:length(cytokines35)){

cor[x,j] = cor(edata[cytokines35[x],],ME_1A[,j])

pcor[x,j] = cor.test(edata[cytokines35[x],],ME_1A[,j])$p.value

}

}

rownames(cor) = cytokines35

rownames(pcor) = cytokines35

colnames(cor) = colnames(ME_1A)

colnames(pcor) = colnames(ME_1A)

module_cytokines = vector(mode = "list")

for (i in 1:ncol(cor)){

module_cytokines[[i]] = rownames(cor)[which(cor[,i] > 0.6 & pcor[,i] < 0.05)]

}

a = 9

cor[module_cytokines[[a]],a]

**#DAVID barplots**

setwd("/Users/alexlandry/Documents/Research/Meningioma/Location/DAVID")

bp = as.matrix(read.delim("turquoise_bp.txt"))

bp = bp[,c(2,11)]

#move bonferroni to 3rd column

bp = cbind(bp,bp[,2])

#change second colunm to process description

bp[,2] = substr(bp[,1],12,nchar(bp[,1]))

#restrict first column to BP term

bp[,1] = substr(bp[,1],1,10)

# P<0.05 only

bp = bp[which(as.numeric(bp[,3]) < 0.05),]

bar_bp = -log10(as.numeric(bp[,3]))

names(bar_bp) = bp[,2]

names(bar_bp)[5] = "peptide/polysaccaride presentation to MHC II"

names(bar_bp)[10] = "exogenous peptide presentation to MHC II"

cc = as.matrix(read.delim("turquoise_cc.txt"))

cc = cc[,c(2,11)]

cc = cbind(cc,cc[,2])

cc[,2] = substr(cc[,1],12,nchar(cc[,1]))

cc[,1] = substr(cc[,1],1,10)

cc = cc[which(as.numeric(cc[,3]) < 0.05),]

bar_cc = -log10(as.numeric(cc[,3]))

names(bar_cc) = cc[,2]

mf = as.matrix(read.delim("turquoise_mf.txt"))

mf = mf[,c(2,11)]

mf = cbind(mf,mf[,2])

mf[,2] = substr(mf[,1],12,nchar(mf[,1]))

mf[,1] = substr(mf[,1],1,10)

mf = mf[which(as.numeric(mf[,3]) < 0.05),]

bar_mf = -log10(as.numeric(mf[,3]))

names(bar_mf) = mf[,2]

kegg = as.matrix(read.delim("turquoise_kegg.txt"))

kegg = kegg[,c(2,11)]

kegg = cbind(kegg,kegg[,2])

kegg[,2] = substr(kegg[,1],10,nchar(kegg[,1]))

kegg[,1] = substr(kegg[,1],1,10)

kegg = kegg[which(as.numeric(kegg[,3]) < 0.05),]

bar_kegg = -log10(as.numeric(kegg[,3]))

names(bar_kegg) = kegg[,2]

bar = c(bar_bp[1:5],bar_cc[1:5],bar_mf[1:5],bar_kegg[1:5])

par(mar = c(5,10,1,1))

barplot(rev(bar),horiz = TRUE, las = 2,col = c(rep("darkgreen",5),rep("red",5),rep("blue",5),rep("black",5)), xlim = c(0,20), cex.names = 0.5, cex.axis = 0.8, xlab = "-log(p)")
